# Draft Genome Sequence of the Heavy-Metal-Tolerant Marine Yeast *Debaryomyces hansenii* J6

**DOI:** 10.1128/genomeA.00983-16

**Published:** 2016-09-15

**Authors:** Camille A. Berrocal, Ramon E. Rivera-Vicens, Govind S. Nadathur

**Affiliations:** Department of Marine Sciences, University of Puerto Rico, Mayagüez, Puerto Rico

## Abstract

*Debaryomyces hansenii* J6 is a heavy-metal-tolerant, flavinogenic yeast isolated from a Swedish estuary. We present here the 11.63-Mb genome of this organism containing 5,717 open reading frames. Comparison with available *Debaryomyces* genomes demonstrated that J6 is closer to *D. hansenii* MTCC234 than *D. fabry* CBS789 and *D. hansenii* CBS767.

## GENOME ANNOUNCEMENT

*Debaryomyces hansenii* is a unicellular, halotolerant, heavy-metal-resistant ascomycete yeast widely distributed in nature (1, 2). This organism has been taxonomically differentiated into *D. hansenii* and *D. fabryi* (3, 4). J6 is a flavinogenic *Debaryomyces* strain that grows between 15 and 37°C and tolerates heavy metals such as cobalt(II) (5).

Genomic DNA was extracted by the method of Cryer et al (6). A library was prepared utilizing the Illumina Nextera DNA sample prep kit, generating 500-bp fragments. Paired-end sequencing (2 × 300-bp) was performed using the Illumina MiSeq platform at Ciris Energy Inc. (Colorado, USA), generating 4,386,465 reads. Quality assessment and trimming of the reads were performed with FastQC (7) and the FASTX-Toolkit (http://hannonlab.cshl.edu/fastx_toolkit/index.html), respectively. *De novo* genome assembly was performed with ABySS version 1.9.0 (8) (*k* = 155), generating 444 contigs after filtering (minimum length 200 bp), for an 11.63-Mb total genome length. Assemblage quality was examined by QUAST (9) (*N*_50_ of 186,435 bp, GC content of 35.43%). Using tRNA-Scan_SE (10) and the WebMGA server (11), a total of 223 tRNAs and three rRNAs (5S, 18S, and 28S rRNAs) were found, respectively. Gene prediction and annotation were performed by the MAKER pipeline (12), which resulted in a total of 5,717 genes. Comparing these results to the transcriptome analysis under cobalt stress for J6 (13) demonstrated that only 998 genes were not expressed under those conditions.

J6 was compared to the MTCC234 (AHBE01000000) (14), CBS767 (NC_006043.2 to NC_006049.2) (15), and CBS789 (LMYN01000000) (16) genomes. Bowtie 2 (17), Burrows–Wheeler alignment (BWA) (18), BBMap (19), and CUSHAW2 (20) were used to align the sequenced reads to the previously mentioned species. Qualimap was used to calculate correctly mapped reads to the genomes (21). The mapping alignment percentages of J6 to the other genomes, depending on the program used, are as follows: (a) MTCC234—95.89% (Bowtie 2), 98.85% (BWA), 96.43% (BBMap), and 98.84% (CUSHAW2); (b) CBS767—13.87% (Bowtie 2), 73.12% (BWA), 48.31% (BBMap), and 80.4% (CUSHAW2); and (c) CBS789—53.08% (Bowtie 2), 88.92% (BWA), 67.94% (BBMap), and 54.68% (CUSHAW2)**.** These results place J6 closest to MTCC234, followed by CBS789 and CBS767.

### Accession number(s).

This whole-genome shotgun project has been deposited at GenBank under the accession number LZDI00000000. The version described in this paper is the first version, LZDI01000000.
